# How can school-based interventions reduce health inequalities? Results from two reviews

**DOI:** 10.1093/eurpub/ckac129.161

**Published:** 2022-10-25

**Authors:** I Moor, A Schneider, L Niederschuh, K Winter, M Richter

**Affiliations:** 1 Martin-Luther-University Halle-Wittenberg, Institute of Medical Sociology, Medical Faculty, Halle, Germany; 2 Technical University of Munich, Department of Sport and Health Sciences, Munich, Germany

## Abstract

**Background:**

Even in young people the chances to grow up healthy are unequal, depending on their socioeconomic position (SEP). In order to reduce these health inequalities, the school is an important field of action for health promotion. However, the evidence is limited regarding interventions focusing on health inequalities. Thus, the aim of the current research is to investigate 1) which school-based interventions contribute to the reduction of socio-economic inequalities in health and health behaviour of children and adolescents and 2) how and under what conditions they are successful.

**Methods:**

A systematic and a realist review were conducted. Some steps of the methodological approach were used synergistically for both reviews: development of the search strategy, selection of the databases (MEDLINE, SSCI, SCIE, DoPHER and TRoPHI) and some inclusion and exclusion criteria. The search covered the years 2000-2020. The screening and subsequent steps were applied specific to each review design.

**Preliminary results:**

The search resulted in 10,524 hits of which 37 publications were included for the systematic review. Most of the interventions focused on nutrition (14), followed by mental health (8) and substance use (5). The results indicate that structural preventive interventions are more likely to reduce health inequalities compared to behavioral preventive interventions. For the Realist Review 7 studies were included. Intrapersonal, interpersonal, and institutional factors were extracted that are relevant for school-based interventions focusing on adolescents with low SEP.

**Conclusions:**

The systematic review showed that school-based interventions are able to reduce health inequalities, but also to increase them. Structural preventive measures seem to be helpful in increasing health equity. The Realist review identified mechanisms of interventions which help to address students with lower SEP.

**Key messages:**

• The evidence regarding interventions with focus on health inequalities is limited. We present two reviews analysing what interventions are needed and how and why they work.

• Structural preventive measures seem to be helpful in increasing health equity. Factors on the intrapersonal, interpersonal, and institutional level are important to address students with low SEP.

